# Comparing barriers to employee assistance program utilization in Canada and the United States using natural language processing and machine learning

**DOI:** 10.1371/journal.pmen.0000589

**Published:** 2026-04-03

**Authors:** Sana Siddiqui, Javier Mencia Ledo, Raihana Premji, Hong Ki Chloe Lau, Kishana Balakrishnar, Charlene Choi, Paula Allen, Allison Kelly, Marilyn Grand’Maison, Donia Obeidat, Ali Bani-Fatemi, Behdin Nowrouzi-Kia

**Affiliations:** 1 Department of Occupational Science and Occupational Therapy, Temerty Faculty of Medicine, University of Toronto, 500 University Avenue, Toronto, Ontario, Canada; 2 TELUS Health, 25 York Street, Toronto, Ontario, Canada; 3 Rehabilitation Sciences Institute, Temerty Faculty of Medicine, University of Toronto, 500 University Avenue, Toronto, Ontario, Canada; 4 Department of Rehabilitation Sciences, Faculty of Applied Medical Sciences, Jordan University of Science and Technology, Irbid, Jordan; 5 Krembil Research Institute-University Health Network, 60 Leonard Ave, Toronto, Ontario, Canada; 6 Institute for Mental Health Policy Research, Center for Addiction and Mental Health, 250 College St, Toronto, Ontario, Canada; National University of Singapore, SINGAPORE

## Abstract

Employee Assistance Program (EAP)s provide critical mental health support, yet utilization remains low in both Canada and the United States. Although qualitative studies have examined general barriers to EAP use, few have compared country-specific perceptions. This study explores barriers to EAP usage and explores potential differences in barriers between Canadian and American workers using natural language processing (NLP). This mixed-methods study, involving data transformation, included 30 semi-structured interviews with EAP-eligible employees (aged 18–65 years) in Canada and the US who had not previously used EAP services. Interviews were analyzed thematically using an inductive approach in alignment with Braun and Clarke’s Thematic Analysis. The qualitative portion of this study was reported in accordance with the Consolidated Criteria for Reporting Qualitative Research (COREQ) guidelines. In Phase 2 of the study, a BERT-based NLP model was trained to classify qualitative quotes by country. In phase 1, three core themes were observed: (1) Structural, informational and systematic barriers influencing EAP accessibility and coverage; (2) Reluctance to use EAP services due to psychosocial and perceptual barriers; and (3) Service quality, scope and cultural relevance to those with multiple social identities. While most barriers were common across countries, US participants reported financial concerns exclusively. Comparatively, in the NLP analysis, financial accessibility and comprehensive support through diverse EAP offerings were important in classifying American quotes, while stigma, vulnerability, and fear of workplace judgement and perceived quality and cultural relevance of EAPs were important in classifying Canadian quotes. Although many barriers to using EAPs are shared, financial and perceptual differences exist between Canadian and American workers. EAPs may benefit from implementing country-specific strategies. This could include creating strategies reducing stigma and quality concerns in Canada, while addressing concerns about cost and alternative options in the United States. Further studies should focus on generalizability and explore sector-specific and longitudinal trends in EAP perceptions.

## 1. Background

Employee assistance program (EAP)s comprise a wide range of resources provided by employers for their employees, and are usually free of charge [1,2]. These services address a broad spectrum of personal and workplace challenges, such as occupational stress, financial struggles, work-life imbalance, and mental health issues. EAPs are designed to improve well-being and, in turn, boost workplace productivity, reduce absenteeism, and enhance job satisfaction [2,3]. Several previous studies have confirmed their effectiveness [3,4]. Despite the numerous benefits that EAPs offer, current trends indicate underutilization of these programs, highlighting potential barriers to access and utilization. For example, a study by Moore et al. found that many employees reported not using EAPs due to difficulties accessing services, the use of external services, a lack of awareness of the programs, and a lack of motivation to use them [5]. Other studies have reported similar barriers, including stigma, doubts about the quality of care, concerns about confidentiality, and administrative barriers, such as long wait times [5,6].

EAPs in Canada and the United States (US) have several similarities and differences. Firstly, the nature of each country’s healthcare system differs. In Canada, the healthcare system provides all citizens with access to basic healthcare services [7]. For example, the Ontario Health Insurance Plan is available to all residents of the province and provides partial or full coverage for doctor and hospital visits, dental services, optometry services, ambulatory services, and more [8]. Conversely, the United States operates a hybrid model that relies heavily on private insurance. As of 2019, nearly 50% of Americans depended on employer-sponsored or private insurance to access healthcare services [9]. This divergence means that in the US, EAPs may serve as a critical supplemental resource to fill gaps in insurance coverage, whereas in Canada, they may act more as a complement to public services. These systemic differences can influence the scope and perceived necessity of EAPs in each country.

Interestingly, despite the outlined differences between Canada and the United States, utilization rates remain low, and barriers to access and usage remain similar across the two countries [5,10]. For example, an overview of EAPs in Canada conducted in 2012 found that utilization rates were as low as 1.2% [11]. Similarly, Taranowski and Mahieu found that utilization of EAPs was as low as 6% in the United States [12]. Canada and the United States are also similar in their delivery of EAP services, with a consistent transition towards telehealth services in recent years; increasing the access to healthcare services both in-person and online [13]. Although EAP utilization is similarly low in both countries, the literature has yet to establish whether Canada and the United States differ in why EAPs are not used. This may help us understand specific cultural nuances that may not be evident at first glance. While individual studies evaluate EAPs in each country, there are limited studies that comprehensively compare these differences. Our study’s findings may help further inform promotion strategies to increase EAP utilization rates. Natural Language Processing (NLP) offers promising avenues to conduct such comparative analyses. By leveraging NLP techniques, it becomes possible to systematically analyze participants’ language and identify patterns in perceived barriers that may be culturally or systemically influenced.

NLP is a subfield of artificial intelligence (AI) concerned with the interaction between computers and human (natural) languages [14]. It enables machines to read, interpret, and derive meaning from human language in a manner that simulates comprehension. NLP has become increasingly integrated into everyday technologies, such as virtual assistants, spam filters, and machine translation tools [15]. At its core, NLP employs computational algorithms and statistical models to analyze textual data, enabling tasks such as syntactic parsing, semantic analysis, and entity recognition [14]. One particularly powerful application of NLP is sentiment analysis, which interprets the emotional tone or polarity embedded in written language. By examining linguistic cues such as grammar, sentence structure, and word choice, sentiment analysis can infer attitudes, opinions, and emotional valence [16]. In qualitative studies, it is increasingly being used to generate thematic codes, allowing for efficiency and more analytical depth [14,17].

There are several frameworks within NLP, each designed to interpret human language in distinct ways. One of the most influential is the Bidirectional Encoder Representations from Transformers (BERT) model, an open-source machine learning architecture developed by Google [18]. BERT enables computers to understand the context of ambiguous language by analyzing the surrounding text in both directions—before and after a given word, allowing for a more sophisticated interpretation of meaning [18]. This bidirectional approach surpasses earlier unidirectional models and supports a nuanced comprehension of language. Specifically, BERT can also be applied to predict contextual information from qualitative quotes, such as identifying a speaker’s country of origin based on linguistic cues. This capability makes BERT a powerful tool for analyzing narratives of differences across countries, particularly when examining service utilization patterns or motivations.

To date, no known studies have combined qualitative data from both Canada and the United States to explore barriers to EAP usage. Additionally, NLP techniques have not been previously used to classify or predict participants’ country of origin based on their perspectives on EAPs. These techniques may enable further interpretation of specific linguistic differences in quotes. This study fills that gap through a novel approach with two primary objectives: (1) to explore the barriers employees face in accessing EAP services in Canada and the United States, and (2) to explore country-specific patterns in these barriers through NLP-driven thematic and linguistic analysis.

## 2. Materials and methods

### 2.1 Ethics Statement

This study was approved by the University of Toronto’s Research Ethics Board (Protocol Number: 42260). Participants provided informed consent electronically after reviewing the study procedures and confidentiality protocols. They were informed of their right to withdraw from the study at any time.

### 2.2. Study design

**Fig 1:**
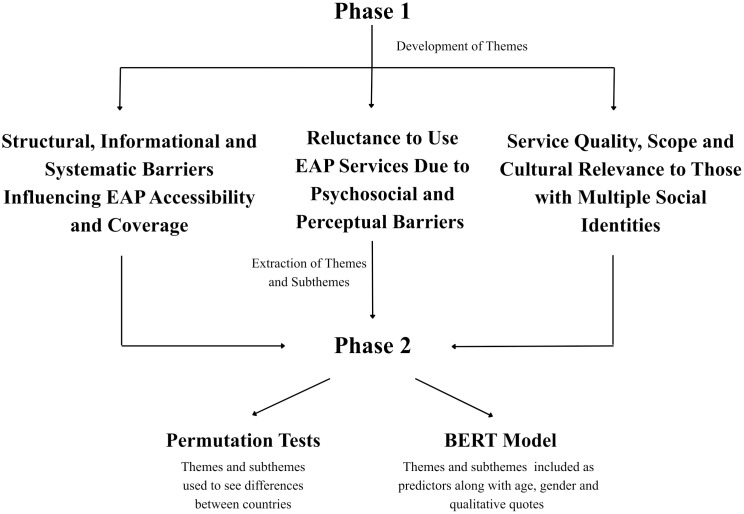
Flow Diagram of Phases.

This study used an atypical mixed-methods approach, with data transformation of qualitative data [19]. Phase 1 involved qualitative data collection via semi-structured interviews to identify key barriers and facilitators to EAP use. An inductive thematic analysis was used to develop a coding framework and extract illustrative quotes. These quotes served as the foundation for Phase 2, which involved a conceptual NLP analysis. In this phase, a BERT model was trained to predict the country of origin of each quote based on its linguistic content. This design enables the exploration of user perspectives in the first phase and statistical validation and classification through machine learning in the second phase. The qualitative results directly informed the NLP model development, ensuring coherence between the phases and enabling a rich, contextually grounded analysis of cross-national barriers to EAP utilization [20,21]. Please see Fig 1 for a flow diagram of the linkage between Phase 1 and 2.

### 2.3. Phase 1: Qualitative Study

#### 2.3.1. Study design.

This study employed a qualitative descriptive design to examine the enablers and barriers to EAP use. One-on-one interviews, averaging 60 minutes each, were conducted via Microsoft Teams with participants between March 3 and 25, 2025. Participants were asked about their knowledge, exposure to, and use of EAPs, as well as their current mental well-being, including their support network, level of happiness, and job satisfaction. The qualitative portion of this study was reported in accordance with the Consolidated Criteria for Reporting Qualitative Research (COREQ) guidelines [22] [see S1 Checklist].

#### 2.3.2. Research team and reflexivity.

Interviews were conducted by RP, KB, and CC, three trained female research assistants. The interviewers were female based on availability to work during the recruitment period. They all had prior qualitative experience and academic backgrounds in population health and psychology. All three interviewers attended an orientation session with a major global EAP provider to gain a deeper understanding of EAP systems before data collection. No prior relationships were established between the interviewers and participants.

#### 2.3.3. Participant selection.

Potential participants were recruited through purposive sampling and identified through a third-party vendor using recruitment criteria. This approach was used to readily access individuals that fit the inclusion criteria. Participants were eligible for this study if they were between the ages of 18 and 65, resided in Canada or the United States, spoke fluently in English, were currently employed (full-time or part-time), and had not utilized an EAP before. A list of eligible participants, along with their contact information and availability, was then shared with the principal investigator (BNK) to facilitate interview scheduling. The final sample consisted of 30 participants. There were 15 participants from Canada and 15 participants from the United States. The sample consisted of 15 females and 15 males. This sample size was deemed sufficient for data saturation, as the 15th interview in each country consistently repeated general themes and ideas. This also aligns with previous literature, that have deemed sample sizes between 5–30 sufficient for detecting themes in qualitative research [23,24]. All participants were between 18 and 65 years old. These participants consisted of full-time and part-time employees.

#### 2.3.4. Setting.

Interviews were conducted virtually through Microsoft Teams. Participants could join interviews from any setting of their choice. No other individuals, except the interviewer and participants, were present.

#### 2.3.5. Data collection.

For participant recruitment, demographic information was collected in advance by a third-party vendor to ensure a balance between Canadian and American participants. Additionally, gender representation was accounted for to include an equal number of male and female participants, however, non-binary and gender-diverse individuals were also eligible for this study to ensure representation. Canadian and United States participants were not matched samples. All interviewers followed a standardized script developed in advance by the principal investigator (BNK) (See S1 File). This ensured consistency between the interviews. Interviews were conducted once with each participant, and no repeat interviews were conducted. Interviews were video recorded with participants’ consent. If consent to video recording was not given, detailed notes were taken instead. If interviews were video-recorded, no additional field notes were taken during or after the interviews. Recordings were then reviewed for situational interpretation following the interview stage. Although transcripts and study findings were not returned to participants for comment, they were made available at the participant’s request.

#### 2.3.6. Data analysis.

Braun and Clarke’s six stage thematic analysis informed the data analysis process [25] Phase 1–3: Interviews were transcribed using NVivo 14 [26], and transcripts were reviewed independently by research team members (BNK and ABF). Three coders (RP, KB and CC) helped generate codes. Descriptive codes were first generated through an initial examination of the text from each interview to capture themes. To ensure inter-rater reliability and to develop initial patterns, each coder independently open-coded 25% of the transcripts. Coders then discussed the findings and compared their codes to ensure consistency. Coding reliability was established at about 77% agreement, which met the absolute agreement level [27]. Any conflicts were reviewed, in joint team meetings with the senior responsible author, BNK. The remaining transcripts were then coded using NVivo 14. Codes based on barriers to EAP use and country differences were used to inform the creation of themes. Specifically, this analysis employed an inductive approach, allowing themes to emerge without preconceived ideas or hypotheses. Phase 4–6: Themes were then reviewed and revisited to ensure they aligned with the generated codes. Detailed and salient quotes were selected to use for the final report.

#### 2.3.7. Trustworthiness and Rigor.

Trustworthiness and rigour were established through a collaborative, structured analytical approach. Researcher triangulation was ensured through the use of multiple coders throughout the process, thereby decreasing the potential for individual biases. To reduce interviewer bias, interviewers were trained to ensure they were meeting standard practices. A critical examination of the data was conducted throughout the process, and researchers maintained an audit trail to enhance transparency. Research members with experience in occupational science and therapy contributed to the development of this manuscript to ensure that the findings were scientifically supported and aligned with occupational health practices. Interview data, including recordings and transcripts, was stored on encrypted, password-protected servers at the University of Toronto. Access to the data was restricted to authorized members of the research team.

### 2.4. Phase 2: NLP analysis

#### 2.4.1. Study design.

To ensure ethical considerations for AI analysis were met, strict anonymization procedures were taken. All personal identifiers were removed, apart from general demographic information, including age and gender. Phase 2 of this study formulated a classification problem through conceptual analysis, informed by NLP techniques and anonymized quotes from Phase 1. Quotes were selected based on the themes identified in the first part of this study. A total of 295 quotes were derived from the 30 participants, of which 15 came from each country. A total of 155 quotes were from Canada, and 140 were from the United States. In particular, 166 quotes were from female participants, while 129 were from male participants We then employed an ensemble model and a reduced dataset obtained by matching demographic variables across the two countries to classify quotes with Propensity Score Matching (PSM) and identify key barriers to EAP usage for each one [28,29]. The primary goal of this phase was to determine if the quotes could be used to identify the participant’s country of origin. The predictors included the quote, the assigned theme and sub-theme, and demographic variables such as age and gender. While the participant count is small, the use of NLP is justified by the relatively high volume of discrete text units (N = 295 quotes), which allows for a more granular, computational analysis of linguistic subtleties than manual coding alone. Furthermore, integrating NLP with PSM and an ensemble model provides a rigorous, reproducible framework to control demographic covariates and minimize researcher bias when identifying country-specific barriers to EAP usage.

#### 2.4.2. Variables.

Both age and gender were identified as potential confounders, but their effect was mitigated through PSM. The only quantitative variable, age, was included as a continuous feature in the machine learning model. It was scaled using MinMaxScaler to normalize its impact alongside the categorical variables, thereby scaling it to values between 0 and 1. The matching process specifically used age as a key matching criterion because the initial analysis revealed that the age distributions between the two countries were significantly different, highlighting the potential for age to confound the thematic analysis.

#### 2.4.3. Data sources and measurement.

All quotes were derived from interview data discussed in phase 1. Each quote was classified based on its country of origin and theme. These three themes were 1) structural, informational, and systematic barriers influencing EAP accessibility and coverage; 2) reluctance to use EAP services due to psychosocial and perceptual barriers; and 3) service quality, scope, and cultural relevance to those with multiple social identities (Fig 3). Subthemes for each theme from phase 1 were also considered during the analysis. Age and gender were identified for each quote. This analysis uses NLP techniques (including BERT embeddings, numerical representations of text that capture word meanings in context, and ensemble classifiers) to examine thematic and linguistic differences in quotes and to explain why US and Canadian respondents are not using EAP services. The primary assessment method combines text features (quotes, themes, sub-themes) with demographic variables and evaluates them using cross-validated machine learning. PSM ensures comparability between groups by balancing age and gender distributions, allowing fair comparisons. While assessment methods remain consistent across groups, the matched design guarantees that observed differences reflect true thematic/cultural variations rather than demographic biases.

#### 2.4.4. Bias.

To minimize bias, we randomly split the data into training and test sets using 5-fold cross validation. Given the US and Canadian group differences in age and gender, to ensure a fair and unbiased model, we used PSM to balance age and gender between US and Canadian participants. This helped mitigate demographic confounding and ensured that the classifier focused on thematic and linguistic content.

#### 2.4.5. Study size and sample justification.

From the original sample of 295 quotes, PSM reduced this to 82 well-balanced quotes (41 per country), which ensured impartial comparisons. To mitigate the risks of overfitting and instability inherent in small datasets such as this one, we used a transfer learning approach with a pre-trained BERT architecture, which leverages prior knowledge from massive corpora to maintain stability on niche datasets. We further addressed model reliability by employing an ensemble method and k-fold cross-validation, ensuring that the high accuracy observed was not a result of memorizing noise but rather a reflection of linguistic signals. Despite the reduction in volume, the model’s performance on both the matched and full datasets suggests that the PSM-matched subset retained a high signal-to-noise ratio, providing sufficient statistical power to distinguish country-level nuances while controlling demographic confounding variables.

We mitigated the risk of pseudo-replication by using pre-trained BERT architecture. Since the model uses transfer learning, it requires fewer samples to achieve stability and is less prone to catastrophic overfitting typical of training smaller models from scratch.

#### 2.4.6. Statistical methods.

Qualitative quotes were thematically coded and integrated with demographic data for cross-country analysis. The methodology followed a three-phase pipeline: thematic frequency analysis, demographic balancing, and ensemble classification.

First, proportional frequencies were compared using permutation tests (10,000 iterations) with Bonferroni correction (alpha = 0.05). To address confounding, we used Propensity Score Matching (PSM) (1:1 nearest-neighbor, 0.1 caliper, exact gender matching) to create balanced cohorts of 41 US and 41 Canadian participants. This reduced standardized mean differences for age and gender to <0.1.

For classification, we used an ensemble of models (Logistic Regression, SVM, Random Forest, and XGBoost) with soft voting. Feature extraction employed BERT (bert-base-uncased) to generate 768-dimensional embeddings, concatenated with thematic and demographic features (total vector: 775). The model underwent stratified 5-fold cross-validation. To test generalizability, the final model was evaluated on the full, unmatched dataset (n = 179).

Finally, feature importance analysis was conducted for each component model: permutation importance for Logistic Regression and SVM, Gini importance for Random Forest, and gain importance for XGBoost. While tree-based models (Random Forest and XGBoost) provide non-negative importance by default, directionality was assigned by multiplying these scores by the sign of the Point Biserial Correlation between each feature and the target variable. For each model, importance scores were extracted and interpreted based on their direction, positive values indicating features predicting the United States and negative values indicating features predicting Canada. Results were triangulated with qualitative findings to identify thematic predictors of country classification.

**Fig 2:**
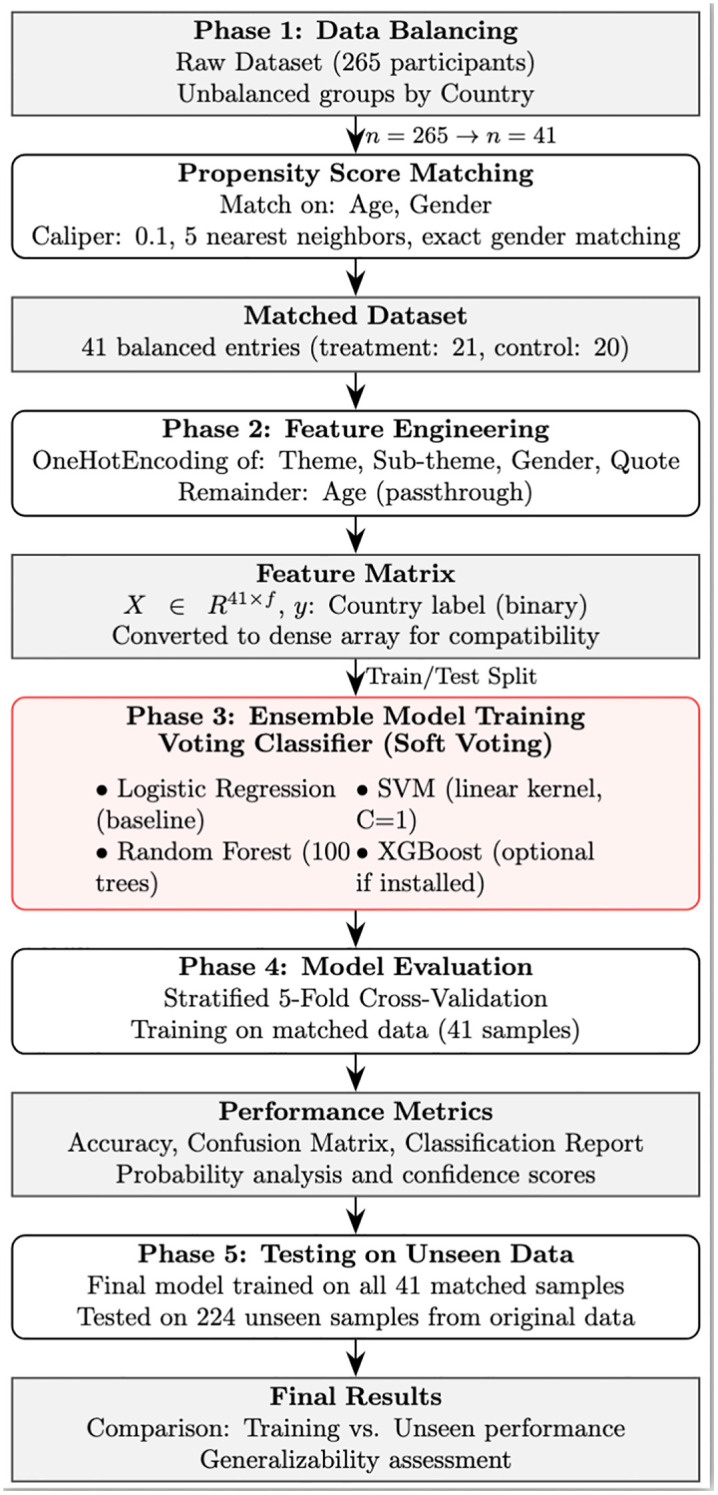
Schematic Diagram of the Study.

All analyses were implemented in Python 3.9 using scikit-learn (v1.2), transformers (v4.28), and xgboost (v1.7) libraries. The complete methodological pipeline is illustrated in Fig 2.

## 3. Results

### 3.1. Phase 1 results

This study included 30 participants: 15 from Canada and 15 from the United States. Fifteen participants were female, while 15 were male. 10 participants were male in Canada, while 10 participants were female in the United States. Thirty participants were spread equally (n = 6) across age groups (18–24, 25–34, 35–45, 46–54, 55–65). Three participants in each age group were from Canada, while three were from the United States. Participant job titles in Canada were classified using the National Occupation Classification (NOC) 2021 broad occupational categories: Business, Finance and Administration Occupations (n = 4); Natural and Applied Sciences and Related Occupations (n = 4); Occupations in Education, Law and Social, Community and Government Services (n = 3); Occupations in Art, Culture, Recreation and Sport (n = 1); Sales and Service Occupations (n = 2); and Trades, Transport and Equipment Operators and Related Occupations (n = 1). Comparatively, participant job titles in the United States were classified using the Dictionary of Occupational Titles (DOT): Professional, Technical, and Managerial Occupations (n = 9); Clerical and Sales Occupations (n = 5); and Processing Occupations (n = 1). A complete summary of participant demographics is presented in Table 1.

**Table 1:** Participant demographics (n = 30).

Participant	Country of Residence	Sex	Age Group	Occupation Group (Code)
P1	Canada	Male	25-34	Natural and Applied Sciences and Related Occupations (2)
P2	Canada	Female	35-45	Occupations in Art, Culture, Recreation, and Sport (5)
P3	Canada	Male	25-34	Natural and Applied Sciences and Related Occupations (2)
P4	Canada	Male	35-45	Natural and Applied Sciences and Related Occupations (2)
P5	Canada	Female	46-54	Sales and Service Occupations (6)
P6	Canada	Female	35-45	Business, Finance and Administration Occupations (1)
P7	Canada	Male	25-34	Occupations in Education, Law and Social, Community and Government Services (4)
P8	Canada	Female	55-65	Business, Finance and Administration Occupations (1)
P9	Canada	Female	18-24	Sales and Service Occupations (6)
P10	Canada	Male	18-24	Business, Finance, and Administration Occupations (1)
P11	Canada	Male	18-24	Occupations in Education, Law and Social, Community and Government Services (4)
P12	Canada	Male	46-54	Trades, Transport and Equipment Operators and Related Occupations (7)
P13	Canada	Male	55-65	Natural and Applied sciences and Related occupations (2)
P14	Canada	Male	46-54	Occupations in Education, Law and Social, Community and Government Services (4)
P15	Canada	Male	55-65	Business, Finance and Administration Occupations (1)
P16	United States	Female	18-24	Clerical and Sales Occupations (2)
P17	United States	Female	18-24	Clerical and Sales Occupations (2)
P18	United States	Male	25-34	Professional, Technical, and Managerial Occupations (0/1)
P19	United States	Male	25-34	Professional, Technical, and Managerial Occupations (0/1)
P20	United States	Male	35-45	Professional, Technical, and Managerial Occupations (0/1)
P21	United States	Female	46-54	Professional, Technical, and Managerial Occupations (0/1)
P22	United States	Female	55-65	Clerical and Sales Occupations (2)
P23	United States	Female	46-54	Professional, Technical, and Managerial Occupations (0/1)
P24	United States	Female	55-65	Processing Occupations (5)
P25	United States	Female	35-45	Clerical and Sales Occupations (2)
P26	United States	Male	46-54	Professional, Technical, and Managerial Occupations (0/1)
P27	United States	Female	25-34	Professional, Technical, and Managerial Occupations (0/1)
P28	United States	Male	35-45	Clerical and Sales Occupations (2)
P29	United States	Female	55-65	Professional, Technical, and Managerial Occupations (0/1)
P30	United States	Female	18-24	Professional, Technical, and Managerial Occupations (0/1)

Three themes were developed from qualitative interviews. These themes, focusing on facilitators and barriers to EAP use, included (1) Structural, informational and systematic barriers influencing EAP accessibility and coverage; (2) Reluctance to use EAP services due to psychosocial and perceptual barriers; and (3) Service quality, scope and cultural relevance to those with multiple social identities.

### 3.2. Theme 1: Structural, Informational and Systematic Barriers Influencing EAP Accessibility and Coverage

Participants discussed ideas related to structural, informational and systematic barriers. Specifically, three subthemes emerged: (1) method of service delivery; (2) awareness and communication; (3) ease-of-use and accessibility and (4) financial accessibility.

#### 3.2.1. Method *of* service delivery.

The method of service delivery was a significant topic of discussion for participants of both countries. Mixed preferences for delivery of EAPs were found. Some participants emphasized the importance of in-person interactions, noting that virtual interactions do not have the same effect. Participants felt they could develop a deeper connection to individuals when interacting in person.

*P2, Canada:…for me in-person means you and the other person has taken the effort and there’s so much more to being in-person. It’s easy to forget a person when you meet them virtually because you meet so many people and everything*

*P18, United States: I feel like with in-person, I’m able to be more transparent with my people. It’s closer, I feel, so it makes me feel closer to the person and I think that would be very helpful.*

Contrastingly, some participants mentioned the ease of online delivery methods, citing convenience and the ability to balance other responsibilities. They felt that an online delivery method could be easier in many ways, including integrating support while managing work hours and family responsibilities, and reducing the need for long drives to mental health services and EAPs.

*P6, Canada: Like if I don’t need to physically go there then, then that would be really helpful. And I can select whatever the time because other than work, I have a family to take care of and kids to take care of.*

*P16, United States: Like, if I have to drive an hour away, just pushes me to not even make that appointment or not get that help just because I have to drive to and back and fit it in my schedule. But if I have my phone, I can do it anytime.*

Some participants also emphasized the importance of hybrid options, which create a balance between delivery methods.

*P15, United States: Maybe a combination of all you know. Online, I probably would start with that... to kind of see what it entails... then maybe look at the materials online... if that’s not working... I may need some help with a professional... then I’d maybe move on to that more one-on-one*

Additionally, participants expressed caution about using AI, feeling that it does not compare to the insight gained from real individuals. Participants addressed concerns about generic and impersonal answers.

*P26, United States: With the chatbot and the self-directed, I’m not a big fan of AI in general for really anything except for productivity stuff like if I need to get help with some calculations I need to make. That’s really all I use it for or repetitive task but for like stuff where I need some sort of customized or creative response, I don’t really trust AI.*

*P3, Canada: Yeah. So, if I wanted to talk to AI chat, I would just go online. Why would I have to reach out to my company? What’s the point of it? Like if I reach out for a service, I much rather a human at the end talk instead of me going to chat for this*

Comparatively, one American participant highlighted the benefits of AI, including alleviating some of the anxiety associated with in-person meetings.

*P16, United States: Chatbot, that’s my favourite one just because it’s just so convenient having it on your phone and like I said, I have anx iety so picking up the phone could be a whole task for me as well, but I know on the Chatbot, I could just text how I text my friends or co-workers. So, it’s just more convenient for me.*

#### 3.2.2. Awareness and communication.

Another barrier is participants’ lack of awareness of EAP services. Participants acknowledged their limited knowledge of the topic, with many stating that they were unfamiliar with the service’s details. Many participants believe that creating awareness and communicating information about EAP services can encourage greater usage. Many participants also personally stated that they would have considered using EAPs if they had more awareness. Canadian and American participants both highlighted limited awareness on EAP services.

*P4, Canada: Never really leaned into how it works and how effective it is. My knowledge regarding this is pretty limited. So might be helpful to me sometimes but I just never knew.*

*P21, United States: Well, I guess it goes back to again, what are EAPs? What falls under EAP services? Because I guess I’m not clear on that aside from mental health and counseling, you know? So I guess if I had a better understanding of what falls under EAP, I’m sure I’d be more likely to use them, and then working for a place that informed employees of their services, that would be great.*

#### 3.2.3. Ease-*of*-use and Accessibility.

Many participants emphasized accessibility as a reason for their lack of EAP usage. Specifically, structural concerns arise, including the lack of EAP information available in one area, lengthy waiting times, and instability with a mental health service provider. These concerns were shared among countries; however, a sole American participant noted reluctance to complete many forms. Participants from both countries also mentioned personal barriers, including a lack of time to seek help.

*P3, Canada: I’ve never used them, but usually one thing that stops me is maybe the wait time. I don’t know what the wait time is going look like. I don’t want be on hold for so long*

*P16, United States: I’m not going to want to fill out 10 different forms and then wait for a call. I feel like just that whole process.*

*P7, Canada: I know this type of platform that you get a different therapist each time, you are not assigned to the same one. So yeah, and I’ll say that could be as good thing or bad thing. I would say some people would just prefer to speak to the same therapist.*

*P23, United States: But the biggest thing for me would be the convenience factor, again I mentioned with trying to seek out therapy in the past, most things happen during the workday. And you want it to be confidential, so you don‘t want to have to tell people why you need this time off*

#### 3.2.4. Financial accessibility.

American participants exclusively mentioned financial concerns. Participants stated they would be open to using EAPs if they were free.

*P18, United States: If it’s free and if it is of better value than solving it on my own, I’m happy to use them*

*P24, United States: If its company paid and I can do it on a virtual or in-person, that would be amazing. I’ll be all for it.*

### 3.3. Theme 2: Reluctance to Use EAP Services Due to Psychosocial and Perceptual Barriers

Participants often did not utilize EAP services due to three psychosocial and perceptual barriers: (1) Mistrust in Confidentiality and Fear of Employer Repercussions; (2) Stigma, Vulnerability and Fear of Workplace Judgement; and (3) Severity for Help-Seeking and Perceived Need.

#### 3.3.1. Mistrust *in* confidentiality and fear *of* employer repercussions.

Many participants expressed concerns about their privacy and the potential sharing of their personal information. Often, individuals had doubts about the confidentiality of their sessions, with many expressing concerns that others at their workplace would discover the information. Additionally, participants cited concerns about accessing an EAP through an employer or HR, not wanting to have to speak to them about their mental health. Lastly, some participants also discussed the difficulties of fully opening up, as they cannot provide confidential information from work.

*P30, United States: I’m not confident about confidentiality. Even if they say it’s private, I worry the employer might find out. That’s why I’d prefer a third-party service.*

*P10, Canada: But I think a hurdle, if I was to use it, would be that I thought you would have to go through a supervisor to do it, which not everybody would want to do. They don’t want to talk things out with their supervisor.*

*P7, Canada: if the conversation related to confidential that stuff, I won’t be able to talk about it. Mostly I’m just hitting the bushes and suggesting that I’m experiencing stuff like that, but I cannot directly say this.*

One participant in the United States also discussed confidentiality concerns with the use of AI applications, such as chatbots. Chatbots are computer programs that enable human conversation with users. Chatbots are increasingly using AI techniques like NLP to respond to and understand user questions [30].

*P22, United States* Chat is usually very convenient, but a chat bot implies that you’re not maybe getting the best information… I worry about confidentiality and whether this is being reported to my employer or something like that.

#### 3.3.2. Stigma, Vulnerability and Fear *of* Workplace Judgement.

Participants highlighted the fear of judgement if they decided to access an EAP. Many participants felt that disclosing their feelings to someone might lead to a difference in how they are treated. Many participants discussed the challenges of opening up to their supervisors, as they did not want to appear vulnerable at work. These issues were highlighted among both Canadian and American participants.

*P3, Canada: I kind of considered it, but I didn’t think it would be that useful. Maybe it would create a negative, unreliable image of me as an employee to management? That’s why I didn’t need that much of it.*

*P29, United States: if you were to discuss work stress or workload, I don’t think that’s something that you would definitely speak to your manager about. Things of that nature, I would feel very uncomfortable about that. Or reaching out to HR about things. I think you have to be very careful about things that you discuss.*

#### 3.3.3. Severity *for* help-seeking and perceived need.

Many participants from both countries stated that they did not feel a need to use EAP services at the time. Participants mentioned their hesitance in accessing work resources, believing that their work life should be kept separate from their personal life. One American participant also mentioned that they didn’t know when to seek help or how serious the mental health issue needed to be.

*P2, Canada: So it’s not that I’m against it or something, it’s just I never felt a need. And by God’s Grace, I didn’t feel ever that I was that stressed that I need to acquire that*

*P30, United States: I tend to separate work from my personal life because I want to maintain professionalism.*

*P26, United States: My issue with it is first I’m not sure what degree of seriousness of why you would seek counsel...I’ve always thought of it more as a little bit more of like a crisis management type thing. So that’s one concern, I don’t feel like my issues or questions rose the level that warranted this.*

Many participants also mentioned receiving help through other resources, including family, friends, social support groups, colleagues and HR. Contrastingly, some participants believe it is best to handle their personal issues independently.

*P19, United States: I just don’t feel the need to seek out more support because I already have something established, especially with my mom. I know I’m in a good place with the relationships I have, and if my needs are already being addressed, I don’t see the need to add more. It’s like not wanting to overcrowd the kitchen with more chefs.*

*P12, Canada: …You know, when there is something going on, if it’s not a major issue, then I I’d rather keep it to myself. If it’s not severely affecting me in a negative way at my workplace, then I’d rather just deal with issues myself.*

### 3.4. Theme 3: Service quality, scope and cultural relevance to those with multiple social identities

Participants also discussed the resources offered through EAPs and factors that would reinforce engagement. Specifically, participants talked about: (1) Perceived Quality and Cultural Relevance of EAPs; and (2) Comprehensive Support through Diverse EAP Offerings.

#### 3.4.1. Perceived Quality and Cultural Relevance *of* EAPs.

Many individuals cited concerns about the usefulness of EAP services. Specifically, participants questioned whether mental health providers would be able to fully understand their issues and personality in a short period of time. Some participants were also skeptical of the quality of EAP services, including the training provided to those offering assistance.

*P24, United States: I think somebody that just doesn’t know you doesn’t know your personality or anything about you. They can just, you know, see how you react to things. See how you answer and stuff compared to your friends. They’ve known you for several years.*

*P9, Canada: I would be interested in dressing like the quality of the services and who I’m talking to and where did these people come from? Like I know you mentioned like consultations and stuff, and just to know, like who I’d be having consultations with, where they come from, etc.*

Two Canadian participants also addressed the complexities of belonging to unique identities. They expressed reservations about EAPs being able to cater to their specific needs.

*P2, Canada: I mean, it could be for a couple of reasons, one being that I’m an immigrant. So, I always feel like my situation is very unique and it may be very situational, which may not be able to be understood very well completely by somebody who is in this part of the world and who may not be an immigrant, who may not have a similar race as me.*

*P7, Canada: Or sometimes the cultural differences making this conversation a little bit hard because they don’t really experience what I did in my cultural background.*

#### 3.4.2. Comprehensive Support through Diverse EAP Offerings.

Lastly, participants from both countries discussed some of the EAP resources they would access in a similar way. Many participants mentioned their need for financial advice. Other resources they would access include counselling, nutrition and fitness plans, life planning, legal advice, and career coaching.

*P14, Canada: And the financial help, a lot of my money, I don’t know where it goes and I’m just kind of struggling with keeping up. So, if I was to have that option I would definitely take it and see what type of help I can get.*

*P9, Canada: Yeah. So like the nutrition aspect, I wasn’t aware of that, so I think that would be cool to be able to talk to a nutritionist and learn more about eating healthy P23, United States: If there’s any sort of job skills training, that would be useful as well. But even general workplace performance and how to improve it, that type of thing, I could see as being super helpful. And I think that’s something that I would use because there‘s not really any other place to get that type of thing*

### 3.5. Differences in EAP use among Participants in Canada and Participants in the United States

American participants exclusively mentioned financial concerns. No other major differences were found in the qualitative portion of this study. Some topics were highlighted exclusively by one or two participants from each country. This includes concerns about confidentiality with AI, the cultural relevance of EAPs, reluctance to complete numerous forms, and the benefits of AI use in mental health services However, it is important to consider the small sample size when discussing these differences.

### 3.6. Phase 2 Results

295 quotes from 30 participants (15 from each country) were analyzed. Canadian participants contributed 155 quotes (52.5%), while US participants provided 140 quotes (47.5%). On average, Canadian participants generated 9.8 quotes (SD = 6.8), compared to 9.3 quotes (SD = 7.3) for Americans.

After PSM, the matched dataset had fewer quotes; however, demographic balance in terms of age and gender was achieved (see Table 2).

**Table 2:** Demographic Characteristics of the Sample after Matching.

Characteristic	Canada	United States
**Quotes**		
Total quotes	41	41
Quotes per participant (SD)	4.6 (1.5)	2.9 (1.4)
Unique participants	9	14
**Gender**		
Female	23 (56.1%)	23 (56.1%)
Male	18 (43.9%)	18 (43.9%)
**Age**		
Mean Age (SD)	38.0 (13.3)	38.5 (12.6)
Age range	22-59	22-57

***Note.*** The table presents the pooled demographics of the participants, with each quote attributed to a single participant rather than to unique individuals.

The BERT-based classification model demonstrated strong performance in distinguishing between Canadian and American participants based on their quotes. On the matched dataset (n = 82, with 41 quotes per country after PSM), the model achieved an overall accuracy of 80.5% (95%CI: [69.3%-92.4%]) using 5-fold cross-validation (80–20 splits, obtaining accuracies in each fold of 70.7%, 88.4%, 68.7%, 100%, 79.2%), with balanced performance across both classes (F1-scores: Canada = 0.81, US = 0.79). Similar scores were obtained for precision (81.0%) and recall (80.0%), and the model misclassified the same number of quotes in both countries.

When applied to the unseen, unmatched data (n = 213), the model achieved a similar accuracy (78.9%, 95% CI: 70.2%–86.1%), with strong precision and recall metrics ranging of around 79%. McNemar’s test comparing performance on matched versus unseen data showed no significant difference (p = 0.172). The model showed a slight edge in predicting Canadian quotes (Canadian precision = 0.84 and US precision = 0.74).

To identify the biggest barriers to EAP usage in each country, the sub-themes and themes derived from quotes for each country were examined. In the United States (140 total quotes), the most frequent sub-themes were awareness and communication about EAPs (33 quotes, 23.6%), method of delivery (22, 15.7%), and severity for help-seeking (20, 14.3%). Concerns such as mistrust in confidentiality (16, 11.4%) and comprehensive support (16, 11.4%) were also prominent, while financial accessibility (5, 3.6%) was exclusive to the US. In Canada (155 total quotes), awareness and communication (39, 25.2%) and severity for help-seeking (38, 24.5%) dominated, followed by mistrust in confidentiality (19, 12.3%). Stigma and fear of workplace judgment were cited more in Canada (13, 8.4%) than in the US (4, 2.9%).

The permutation tests showed which of these differences were significant (Fig 4). In the US, comprehensive support (p = 0.026) and financial accessibility (p = 0.043) emerged as more prominent barriers compared to Canada. Conversely, severity for help-seeking and perceived need was the only barrier that was significantly higher in Canada (p = 0.044), with stigma and fear of workplace judgment also showing differences between Canadians and Americans but not reaching statistical significance (p = 0.090), respectively (Fig 4).

**Fig 3:**
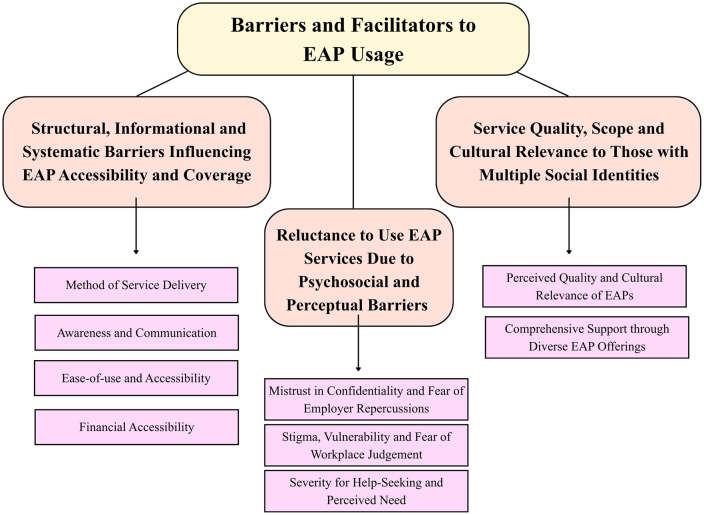
Concept Map.

**Fig 4:**
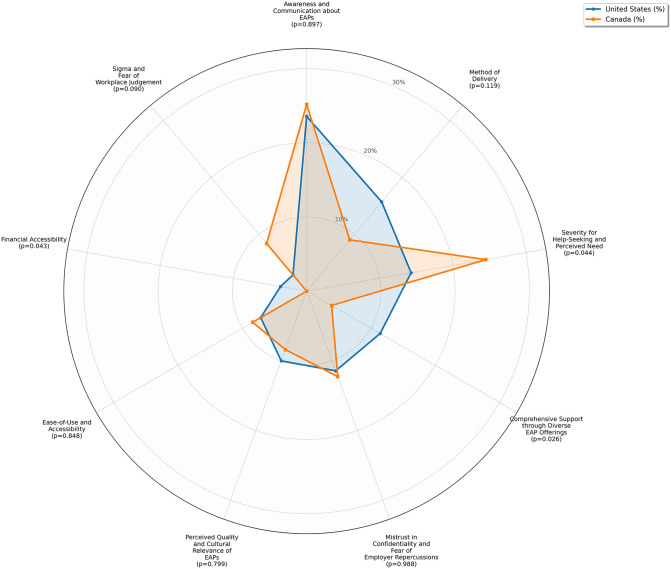
Radar Chart of Quote Sub-theme distribution by country. *Note.* The Radar chart shows the proportion of quotes in each subtheme. Canada is shown in orange and the United States in blue.

The variable importance analysis, derived from four BERT-based classification models (Logistic Regression, SVM, Random Forest, and XGBoost), identified differences in variables that contributed to the classification of American and Canadian participants’ quotes.

For American quotes, the logistic regression placed greater importance on the financial accessibility subtheme (relative importance: 0.844), the availability of comprehensive support through diverse EAP offerings (ri: 0.633) and severity for help-seeking and perceived need (ri: 0.568) in predicting quotes. The random forest and the XGBoost models also highlighted financial accessibility (ri: 0.211 and ri: 0.832 respectively) and comprehensive support through diverse EAP offerings (ri: 0.195 and ri: 0.061 respectively), as well as the method of service delivery (ri: 0.115 and 0.041 respectively), as the key concerns used to identify American quotes. The logistic regression model also assigned importance to quotes related to the lack of awareness and communication about EAPs, highlighting that participants would benefit from “being able to be educated on what the EAP is, what exactly is being offered and what is the added value” (ri: 0.516) and if “there’s an onboarding in terms of what’s being offered as employees” (ri: 0.516).

In contrast, for predicting quotes from Canadian participants, the logistic regression assigned the greatest importance to the subthemes stigma, vulnerability, and fear of workplace judgement (-0.665) and awareness and communication about EAPs (-0.655). The logistic regression also attributed importance to the perceived quality and cultural relevance of EAPs subtheme in classifying Canadian quotes (ri: -0.516). It further highlighted some quotes related to the awareness and communication of EAPs, including “I’ve never really faced any issues like this, but my employer also has a lot of options where we can discuss if there is mental stress, we can always discuss it and they have options like this”. The random forest and XGBoost also assigned the most importance to stigma, vulnerability, and fear of workplace judgment to (ri: -0.153 and –0.026) and severity for help-seeking and perceived need (ri: -0.135 and –0.024) in predicting quotes. The logistic regression also identified Canadian quotes raising concerns about the method of delivery, including “I prefer to do either the in-person I don’t like the chatbot”(ri: -0.526) or “It’s easy to forget a person when you meet them virtually because you meet so many people and everything. So I think that in-person, one to one session, is much more and you can build relationships out of that outside the counseling” (ri: -0.523). Finally, the random forest model also indicated some importance for the subthemes mistrust in confidentiality and fear of employee repercussions and the ease of use and accessibility of EAPs in the classification of Canadian quotes, although with smaller importance magnitudes (ri: -0.048 and –0.053 respectively).

Finally, given the nature of the SVM model, it classified individual quotes to their country of origin with a relative importance of 1 for each quote. The rest of the models showed consistent findings in which concerns themes and sub-themes were driving their predictions of each country.

## 4. Discussion

### 4.1. Summary of key findings

This atypical mixed methods study examined cross-national barriers to EAP use between Canada and the United States. Although results from Phase 1 showed limited differences in EAP usage barriers between countries, Phase 2 results further highlighted observed barrier patterns among countries. Specifically, this study found barriers across three themes: (1) structural, informational and systematic barriers to access; (2) reluctance to use EAP services due to psychosocial and perceptual Barriers; and (3) concern around service quality, scope, and cultural relevance to those with multiple social identities. The combined methodological approach enabled contextual depth, revealing that while many challenges are shared, cultural and systemic distinctions may shape the prominence and framing of these barriers. The qualitative results informed the development of the NLP analysis [20,21].

### 4.2. Theme 1: Structural, Informational and Systematic Barriers Influencing EAP Accessibility and Coverage

This study found that participants in both countries consistently identified method of delivery, awareness, accessibility, and financial concerns as major structural barriers. While some preferences differed, both groups emphasized a desire for flexibility and clarity in how services are presented and accessed.

In terms of the method of service delivery, the results were mixed for participants from both countries, with some highlighting that in-person interactions allow for a deeper connection, while others emphasize the convenience of virtual services. This is consistent with previous research indicating that individuals often prefer in-person mental health services and EAPs over virtual formats due to enhanced communication and relational cues (e.g., facial expressions and vocal tone) [31], while some others prefer virtual communication for treatment because of its convenience and accessibility, particularly for those struggling to attend in-person sessions [32]. It is important to note that there were no significant differences found for method of delivery in the permutation test, indicating the need for further research on whether method of delivery holds as a barrier in confirmatory analyses.

Regarding awareness and communication, the results from the qualitative and permutation tests and NLP analyses collectively suggest that participants from both countries unanimously stated they were unaware of the nature of the EAP service. This is consistent with the literature, as a past study found that few employees had detailed knowledge of how their firms’ EAPs functioned [33]. The results also align with past studies that identified limited awareness as a common barrier to EAP utilization in both countries [5,34]. This suggests that, despite the availability of EAPs, factors such as insufficient outreach and communication about EAP availability hinder awareness and limit utilization, reducing the impact of their potential benefits.

For ease of use and accessibility, participants from both countries frequently cited these factors as key reasons for not using EAPs. This is reinforced by the results of the permutation tests, which found no significant differences between the United States and Canada, suggesting that accessibility concerns are a shared issue among these two countries. This finding is highly consistent with existing literature, which has identified difficulty accessing EAP services as one of the most reported barriers to their use [5]. To make EAPs easier to use, organizations in both the United States and Canada may need to provide clear and simple information in one place, so employees know how to get help. Reducing waiting times and improving provider consistency may also encourage more people to utilize these services when needed. This subtheme was also slightly influential in predicting Canadian quotes, suggesting the need for more research to confirm findings and specific strategies needed.

The last barrier to accessibility and coverage is financial accessibility. Interestingly, our results showed that financial accessibility is a primary concern for American participants, emerging as a prevalent barrier in qualitative results was significant in the permutation test, and the was important in classifying quotes in the NLP analysis. More research is needed to make definitive conclusions; however, one might speculate that differences in national healthcare systems may explain this pattern. In Canada, the government provides universal healthcare, which significantly reduces the cost of healthcare services. In contrast, the United States relies mainly on a private insurance-based system, where employees often bear the cost of insurance themselves or rely on employers [35]. These results, although speculative, may underscore the need for enhanced accessibility, clearer communication, and diverse methods of service delivery to increase the utilization of EAPs in the United States and Canada.

### 4.3. Theme 2: Reluctance to Use EAP Services Due to Psychosocial and Perceptual Barriers

Three barriers were identified in the qualitative portion of this study for theme 2. These barriers included mistrust of confidentiality and fear of employer repercussions; stigma, vulnerability, and fear of workplace judgment; and severity for help-seeking and perceived need..

No significant differences were found between countries regarding confidentiality. Findings highlight that many individuals reported hesitancy to let employers and coworkers know they had accessed an EAP. This barrier remains consistent in literature worldwide, with many studies on EAPs citing confidentiality as a top deterrent to use [5]. In both Canada and the United States, individuals are often skeptical about the potential ties between EAPs and employers [36]. This, in turn, creates distrust, discouraging individuals from discussing their mental health struggles. These findings suggest the need for more third-party EAPs that create trust for all parties, an idea also recommended in the literature [36]. As confidentiality concerns were slightly influential in predicting Canadian quotes, more research is needed.

A second significant barrier was stigma and fear of workplace judgement for participants in both countries. This fear of negative perceptions remains prevalent in the literature, with many individuals believing they will be labelled ineffective workers or weak for accessing an EAP [37]. Moreover, the permutation test revealed that differences in stigma and fear of workplace judgment quotes across countries approached significance. More interestingly, the NLP analysis used stigma and fear of workplace judgement to predict Canadian quotes in some of the models. Although there is no direct literature on the topic, subtle differences in work culture between countries may provide an explanation and further research is recommended. Consequently, this study found that many participants relied on their friends or families to share mental health concerns. This finding is consistent with the literature, which often highlights that participants tend to initially speak to those they trust [38]. This study also found that some participants preferred to keep mental health concerns to themselves, and this is especially common among men in the literature, who prefer to suppress their feelings instead [38]. Many individuals also reported not needing support for EAPs. This remains a significant reason for EAP underutilization in literature [5,10].

Furthermore, the results of the permutation tests observed that barriers surrounding severity for help-seeking and perceived need were cited significantly more often among Canadian participants. One possible reason for this might be the structure of the public healthcare system in Canada [39]. The public healthcare system may lead individuals who usually rely on their physicians to meet mental health needs to associate help-seeking with the severity of symptoms [40]. Individuals might wait until their symptoms worsen before seeking help. It is important to note that severity for help-seeking was important in classifying both Canadian and American quotes in the NLP analysis. This may suggest that further study is needed to determine the implications of the Canadian and the American healthcare systems and whether severity of help-seeking and perceived need barriers differ between countries.

Findings from both phases highlight how confidentiality concerns, stigma, and perceived need remain prevalent barriers to EAP utilization. Subtle yet significant differences were identified across countries, which can inform future research and strategies to increase EAP use.

### 4.4. Theme 3: Service Quality, Scope and Cultural Relevance to Those with Multiple Social Identities

The third theme contained two subthemes: (1) Perceived Quality and Cultural Relevance of EAPs; and (2) Comprehensive Support through Diverse EAP Offerings. Our qualitative analysis aligns with broader literature on mental health support and workplace well-being, highlighting the need for tailored and high-quality services to enhance EAP utilization. Participants reported that the professionalism and quality of EAP services, especially compared to informal support networks, increased their willingness to engage with them. This is consistent with Siddiqui et al., who found that higher perceived workplace support was significantly associated with improved mental health outcomes among healthcare workers [41]. Together, these findings highlight the critical role of delivering high-quality and relevant EP services in fostering employee engagement and promoting mental well-being.

Furthermore, our findings on this theme underscore the importance of offering a diverse range of services. This supports previous research indicating that EAPs function as comprehensive support systems designed to address a diverse range of employee needs, including mental health support and financial aid [42]. Through offering such diverse and relevant services, EAPs are better able to meet the varied needs of the workplace, thereby increasing the likelihood of employees utilizing them. Notably, the NLP analysis relied on quotes related to comprehensive support through diverse EAP offerings, including other services provided by employers, to predict American quotes. The permutation test for the subtheme was also significant. While causality cannot be confirmed due to the small number of quotes used to predict American quotes, systemic differences in healthcare infrastructure may provide one speculative answer to these observed patterns. Those in the United States rely on insurance-based healthcare systems [39]. American employees may prefer mental health services that are explicitly integrated into their insurance coverage, reflecting a reliance on employer-provided benefit systems.

The importance of cultural relevance in strengthening user engagement with health services is also well established in current literature [43–45]. A study by Hom et al. found that cultural incompetence is a key barrier to mental health service utilization, particularly among immigrant and ethnic minority populations in Canada and the United States, who may avoid seeking help due to concerns over their provider not considering their cultural context [45]. Moreover, a systematic review conducted by Khosravi et al. reported that diversity was a strong factor influencing users’ engagement with mental health technologies, stating that inclusivity of various backgrounds allows for consideration of different perspectives, needs, and experiences [44]. Allen also indicated that creating culturally responsive workplace supports can help promote engagement, trust, and mental well-being among its workers, which in turn can lead to lower turnover rates and better workplace outcomes [46].

Our findings also reveal that Canadian participants more frequently expressed these concerns. Canada has a highly diverse population comprising various ethnic groups [47], which underscores the need for workplace support programs, such as EAPs, to be culturally inclusive for its workers. When it comes to service quality and scope, Canadians benefit from universal access to healthcare services [39]. As a result, the quality of workplace support must be high enough to compete with or complement existing healthcare options. This finding is consistent with our results, which suggest that some participants preferred their regular healthcare providers over EAP services, primarily due to the strong rapport built over time. Collectively, these results emphasize the importance of delivering culturally responsive, high-quality EAP services to enhance user engagement and program efficacy.

### 4.5. Limitations

This study is exploratory and as such has several limitations that should be considered when interpreting the results. First, the qualitative data for the NLP analysis was derived from interviews with a relatively small sample size (30 participants), which may limit the generalizability of the findings. However, data saturation was monitored, and ideas were repeated by the 30^th^ interview. No additional field notes were taken during or after the interviews, which may have limited the depth of situational interpretation and reduced opportunities for triangulating findings and may have led to potential demographic or language-based biases. However, interviews were video recorded allowing for situational interpretation that may not have been captured during the interview. Furthermore, researcher triangulation ensured that multiple coders reviewed transcripts and recordings, ensuring individual biases reviewing the data without field notes were mitigated. While the sample was balanced between Canadian and American participants, the small number of quotes per subtheme (e.g., only five quotes on financial accessibility in the US) may not fully capture the breadth of perspectives within each country. However, this risk was partially mitigated by the depth of the semi-structured interviews, which ensured that even less frequent subthemes were grounded in rich, context-specific data

The NLP model, while robust, also has limitations. PSM was used to balance the age and gender distributions between the Canadian and American samples, resulting in a reduced sample size. The model’s accuracy (79.3–85.9%) suggests room for improvement, particularly in distinguishing subtle linguistic nuances between countries; nonetheless, these accuracy rates remain well above the threshold for providing meaningful thematic classification in exploratory NLP research. Furthermore, the BERT model’s reliance on pre-trained language representations may not fully account for cultural or regional dialects that could influence thematic expression. A further methodological consideration is the potential for pseudo-replication, as the 295 quotes were derived from 30 participants. While BERT analyzes quotes as individual sequences, we sought to minimize this risk by ensuring a diverse range of speakers contributed to each theme, thereby supporting the generalizability of the national patterns over individual linguistic idiosyncrasies.

Despite these limitations, the study provides valuable insights into initial country-level patterns in EAP barriers. The findings suggest that while structural barriers (e.g., awareness, delivery methods) are shared across both countries, perceptual and systemic differences exist. Americans’ emphasis on financial accessibility aligns with their private healthcare system, whereas Canadians’ focus on stigma and cultural relevance may reflect broader societal attitudes toward mental health. The NLP analysis successfully identified these patterns, demonstrating the potential of AI to augment traditional qualitative methods.

A cautious interpretation is warranted: the observed differences may stem from other, unmeasured demographic imbalances (e.g., a higher proportion of participants in managerial roles in one country) or other unmeasured cultural factors. However, the consistency between qualitative themes and NLP predictions strengthens the validity of the results. Policymakers and employers should consider these findings when tailoring EAP outreach, such as addressing cost transparency in the US and providing confidentiality assurances in Canada.

### 4.6. Areas of future research

While this study provides meaningful insights into country-level differences in barriers to EAP use among Canadian and American workers, several avenues for future research remain. Future research could enhance the findings of the current study by focusing on more nuanced differences across countries, by considering the impact of cultural values and workplace norms and expectations. Potential French-speaking Canadians were not included in this study to avoid misinterpreting unique linguistic differences in the French language. Future research should look at unique EAP utilization barriers for French-speaking Canadians as well. Furthermore, there may be additional differences across sectors and organizational types that can significantly influence access to and use of EAP services; future research should consider qualitative investigations that compare experiences across sectors (e.g., public vs. private).

Future research would particularly benefit from an intersectional approach to better understand how overlapping social identities shape the employee’s experience with EAPs. In doing so, potential systemic inequities may reveal unique barriers to accessing and using EAPs among underrepresented groups. Additionally, future research would benefit from incorporating managerial and HR perspectives to better understand the organizational dynamics and barriers that influence employee decisions regarding EAP use. Although this study observed EAP differences between Canada and the United States, further differences may exist within each country. Ultimately, future studies should prioritize research in understanding how intersectional approaches may provide employers with tangible insights for delivering inclusive EAPs that support the needs of a diverse workforce. For example, such research might highlight the importance of tailored intervention programs for specific intersecting identities and culturally diverse service providers who are better able to support and address the unique needs of individuals from underrepresented groups.

Future studies should also prioritize larger, more diverse samples to enhance the generalizability of findings, ensuring representation across industries, socioeconomic backgrounds, and linguistic groups. Longitudinal approaches could track EAP perceptions over time, revealing how workplace policies, economic shifts, or healthcare reforms influence usage barriers. Combining NLP with survey data would strengthen quantitative insights, while multilingual models could capture the voices of underrepresented individuals. Additionally, intervention studies, such as testing AI-tailored EAP outreach, could assess whether addressing country-specific barriers improves engagement

### 4.7. Recommendations

Although this study is exploratory, EAPs and policymakers could benefit from implementing culturally tailored EAP promotion and utilization strategies. In Canada, focus can be allocated to reducing stigma and addressing concerns about the quality of EAP services. Interventions may involve mental health education and EAP promotion. In the United States, EAPs may benefit from responding to concerns about cost and the presence of alternative support options. Strategic steps may include transparent approaches to handling costs, more education about in-person EAP access, and promotion strategies, where EAP’s benefits, low financial costs, and availability of different methods of delivery can be shared. Despite these differences, many concerns, including limited awareness, method of delivery, and severity for help-seeking and perceived need, were shared amongst participants from both countries, indicating that joint efforts to address EAP utilization concerns are needed.

## 5. Conclusions

This study provides insight into the potential barriers to EAP usage, including differences in barriers faced by individuals in Canada and the Unites States. Although there are limits to generalizability as findings from this study are exploratory, preliminary differences between Canadian and US participants found in this study may better inform EAP programs and policymakers. Findings from this study demonstrate the need for culturally tailored EAP promotion and utilization strategies. Overall, this study highlights the importance of further examining cultural distinctions and similarities to increasing utilization in both countries. Future research should explore these barriers longitudinally and across sectors to improve generalizability and inform targeted strategies to increase EAP usage.

## Supporting information

S1 Checklist Consolidated Criteria for Reporting Qualitative Research (COREQ) Checklist. (DOCX)

S1 File Supporting Information. Interview Guide.
(DOCX)

S1 Fig Logistic regression: Features driving Canada vs US predictions. (TIFF)

S2 Fig XGBoost: Sub-themes driving country selection. (TIFF)

S3 Fig Random Forest: Sub-themes driving country selection. (TIFF)
