# Supplementary material for: Comparing barriers to employee assistance program utilization in Canada and the United States using natural language processing and machine learning
Source: PLOS Ment Health. 2026 Apr 3;3(4):e0000589. doi: 10.1371/journal.pmen.0000589 (PMC13048493; doi:10.1371/journal.pmen.0000589)
Supplement: S1 File — Interview Guide. (DOCX) [file pmen.0000589.s002.docx]

*Interview Protocol and Questions*

**Before Interviewing the Participant**

Confidentiality is discussed. This includes informing participants that only the research team will have knowledge of what the participant discusses and that the interview will be combined with other interviews for analysis.

The interviewer will request permission to record the interview and explain the importance of recording for preserving the accuracy of data collection.

**Interview Questions**

*Participants who have used EAP services:*

1. Can you describe your overall experience with the Employee Assistance Program (EAP) offered by Telus Health?
2. Can you share with us how you found this EAP and what made you decide to use the service?
3. In what ways, if any, has the EAP positively impacted your overall mental health and wellbeing?
   1. How has the EAP influenced your happiness and job satisfaction?
   2. Do you believe the EAP has made a difference in your work-life balance and overall quality of life?
4. Have you experienced any barriers or challenges in accessing EAP services? If so, what are they, and how could they be improved?
5. Can you provide insights into the level of awareness and understanding of the EAP among your colleagues, and its impact on their mental health?
6. How do you think Telus Health could better promote their EAP and encourage employees to utilize its services?

*Participants who have not used EAP services:*

1. Were you aware of the EAP offered by Telus Health before participating in this study?
2. Have you ever considered using the EAP service before? If not, what factors influenced your decision to not use it? Are there any concerns or reservations you have about utilizing EAP services?
3. Can you describe your current support network for addressing personal or work-related challenges?
   1. How would you describe your current level of happiness and job satisfaction?
   2. In your opinion, what role does mental health and well-being play in your overall job performance and quality of life?
4. If you were to consider using EAP services, what specific challenges or issues would you be interested in addressing?
5. Can you provide insights into the level of awareness and understanding of the EAP among your colleagues, and its impact on their mental health?
6. How do you think Telus Health could better promote their EAP and encourage employees to utilize its services?

*Provide the definition of EAP and a summary of the supports offered by Telus Health*

1. Based on what you now know about EAPs, how do you think the service might complement or enhance your current support network for addressing personal or work-related challenges?
