# Supplementary figures and images for: Comparing barriers to employee assistance program utilization in Canada and the United States using natural language processing and machine learning
Source: PLOS Ment Health. 2026 Apr 3;3(4):e0000589. doi: 10.1371/journal.pmen.0000589 (PMC13048493; doi:10.1371/journal.pmen.0000589)

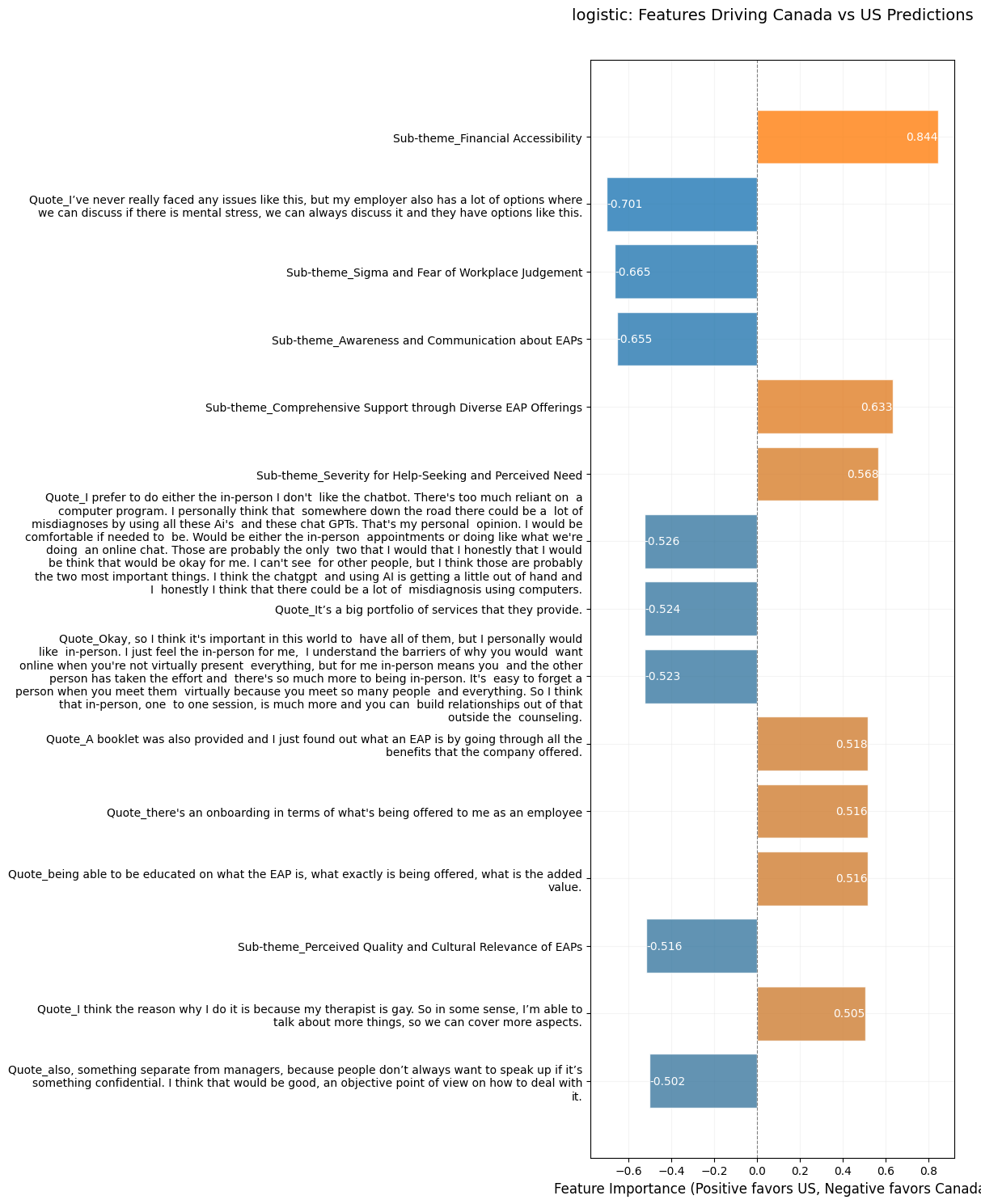

Supplement: S1 Fig — (TIFF) [file pmen.0000589.s003.tiff]

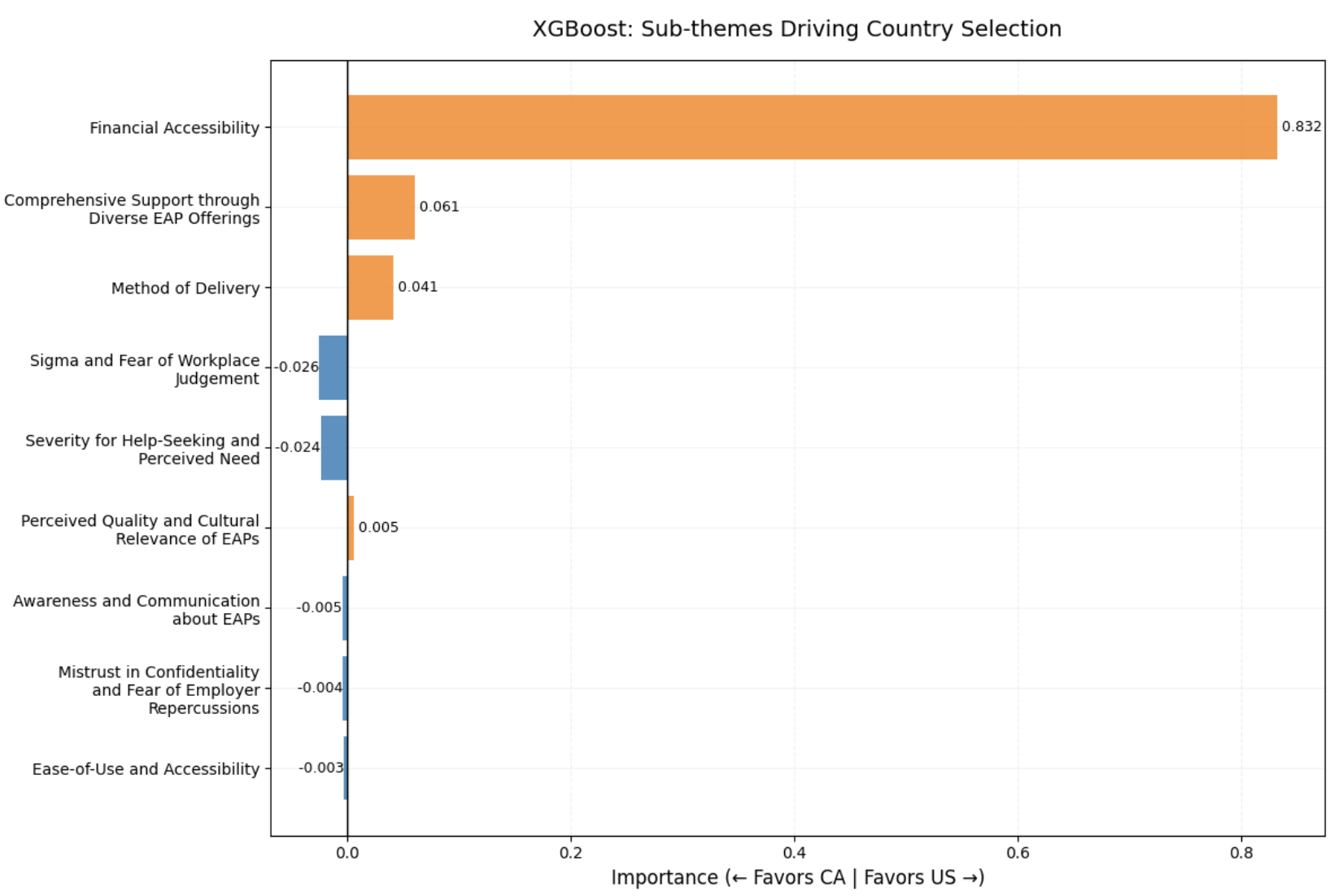

Supplement: S2 Fig — (TIFF) [file pmen.0000589.s004.tiff]

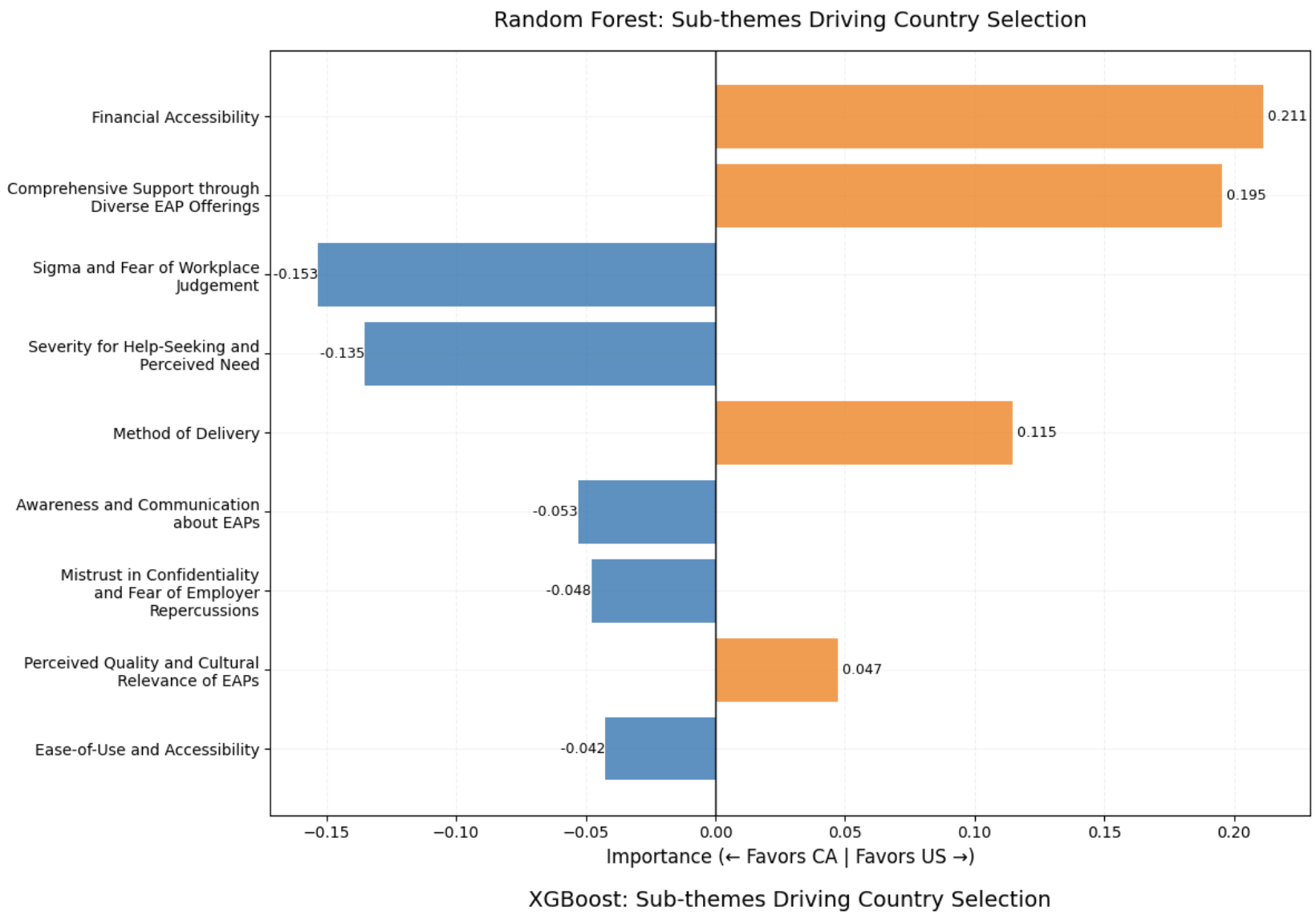

Supplement: S3 Fig — (TIFF) [file pmen.0000589.s005.tiff]
